# Correction: Shi et al. Study on Modification and Mechanism of Construction Waste to Solidified Silt. *Materials* 2023, *16*, 2780

**DOI:** 10.3390/ma19204422

**Published:** 2026-10-21

**Authors:** Yannan Shi, Haoxuan Weng, Jiongqi Yu, Yongfan Gong

**Affiliations:** 1 Zhejiang Guangchuan Engineering Consulting Co., Ltd., Hangzhou 310020, China; 2 Zhejiang Provincial Key Laboratory of Water Resources Disaster Prevention and Mitigation, Hangzhou 310020, China; 3 College of Architectural Science and Engineering, Yangzhou University, Yangzhou 225127, China

The journal’s Editorial Office and Editorial Board are jointly issuing a resolution and removal of the Journal Notice linked to this article [1], as well as an update to the original publication. Following concerns raised about the integrity of the peer review, the Editorial Office has conducted a post-publication peer review of this article. This process included the recruitment of a new independent reviewer, and it was supervised by an Editorial Board member to ensure full compliance with MDPI’s Editorial Process (https://www.mdpi.com/editorial_process).

As a result of this process, the Editorial Board member, and the authors have agreed to update the following aspects of this publication:

- One new report was uploaded to the peer review record (https://www.mdpi.com/1996-1944/16/7/2780/review_report).
- There was an update to the Academic Editor. The Editorial Board member (Prof. Dr. Zbyšek Pavlík) who conducted this post-publication review has been added.
- In Abstract, the sentence “while mixing different grain diameters and quantities of building waste materials and ordinary sand” has been changed to “while mixing construction waste debris”; the word “building” has been changed to “construction”; the sentences “It reduced the content of large pore size of solidified sediment and optimized the internal pore structure. At the same time, it formed a new structure filled by rigid skeleton material.” have been changed to “Addition of construction waste debris reduced the content of large pore size of solidified sediment and optimized the internal pore structure. At the same time, construction waste debris formed a new structure filled by rigid skeleton material.”
- In Section 1, paragraph 1, the sentence “ecological environmental material” has been changed to “ecological environmental friendly material”; the phrase “ecological slope stabilization” has been changed to “slope stabilization”.
- In Section 1, paragraph 2, the citation “[15,16]” has been changed to “[15]”; “[17,18]” has been changed to “[16,17]”; “[19]” has been changed to “[18]”; and “Xu [20–22]” has been changed to “Jalal [19,20]”. The sentence “At present, the solidified soil still has poor water stability and is easy to disintegrate in the dry–wet cycle” has been changed to “At present, the solidified soil still has poor water stability and is easy to disintegrate in the dry–wet cycle [21].”
- In Section 1, paragraph 3, the sentence “this paper proposes to replace part or all of the building waste debris such as sintered brick slag and waste concrete wall as the skeleton matrix of the solidified dredging sludge material.” has been changed to “this paper proposes to utilize construction waste debris as the skeleton matrix of the solidified dredging sludge material.”
- In Section 2.1, the phrase “Geo-technical Test Specification (SL 237-1999)” has been changed to “highway geotechnical Test (JTG 3430-2020) [22]”; “building waste (S)” has been changed to “construction waste (S)”. The sentence “The building waste was mainly of a small particle size, which was 8.63% below 0.075 mm, 78.86% below 0.075 mm and 12.51% above 2 mm.” has been changed to “The construction waste consisted predominantly of fine particles: 8.63% were smaller than 0.075 mm, 78.86% ranged between 0.075 mm and 2 mm, and 12.51% were larger than 2 mm.”
- In Sections 2.2.1, 2.2.3–2.2.6, the descriptions of all methods have been updated to following version:

2.2.1. Unconfined Compressive Strength Test

Thirteen equal portions of sludge, each of a predetermined mass, were prepared and cleared of impurities. Subsequently, varying proportions of solidifying agents, construction waste fragments, or ordinary river sand were incorporated into each portion, with the dosages calculated based on the dry soil mass. The specific mixing proportions are presented in Table 1. The compaction procedure was conducted in accordance with the heavy compaction test specified in the highway geotechnical Test (JTG 3430-2020), with a unit volume compaction effort of 2684.9 kJ/m3. A small three-way compaction mould, measuring 37.5 mm in diameter and 76.4 mm in height, was employed. Each mixture was thoroughly homogenized and then air-dried until the moisture content reached 40%. The prepared material was placed into the compaction mould in three equal layers. Each layer was compacted by 100 free drops of the compaction hammer. After the compaction of each layer, the surface was scarified using a geotechnical knife prior to the addition of the subsequent layer. Upon completion of compaction, the sample surface was leveled with a scraper, wrapped with a sealing film, and cured in a standard curing chamber maintained at a temperature of 20 ± 2 °C and a relative humidity of 95% ± 3%for a period of 7 days.

2.2.3. Permeability Test

The water resistance of solidified sludge was studied by the mass loss and volume shrinkage of samples after standard curing for 7 days after drying and wetting cycles. To simulate dry–wet cycling, the solidified sludge samples were first dried at 40 °C for 24 h, followed by immersion in distilled water for 24 h. Upon completion of the final cycle, the samples were removed from the distilled water and allowed to drain for 1 h prior to weighing. The mass loss of the solidified sludge was subsequently recorded after each designated number of dry–wet cycles. The experimental methods were quoted by the specification for highway geotechnical Test (JTG 3430-2020).

2.2.4. X-ray Diffraction Spectrum

For X-ray diffraction (XRD) analysis, approximately 10 g of the parallel specimen, originally prepared for the unconfined compressive strength test, was collected. The sample was initially dried in a forced-air oven at 40 °C, then pulverized and sieved through a 200-mesh screen. The mineralogical composition of the sieved solidified sludge was subsequently determined by a D8 Discover diffractometer (Bruker Instruments, Ingelfingen, Germany).

2.2.5. Pore Structure

For mercury intrusion porosimetry (MIP) analysis, the parallel specimen from the unconfined compressive strength test was vertically divided into upper, middle, and lower sections. From each section, approximately 10 g of blocky fragments, measuring roughly 7 mm in size, were collected. These fragments were then dried in a forced-air oven at 40 °C for 24 h. Subsequently, the pore structure distribution of the dried samples was determined using an AutoPore IV 9520 mercury intrusion porosimeter (Micromeritics, Norcross, GA, USA).

2.2.6. SEM

For scanning electron microscope (SEM) analysis, a block-shaped specimen measuring approximately 10 mm in size was cut from the parallel sample used in the unconfined compressive strength test. The specimen was initially dried in a forced-air oven at 40 °C for 24 h, after which its surface was sputter-coated with platinum to enhance conductivity. The micromorphology of the prepared block was then examined using a field-emission environmental scanning electron microscope (FEI Quanta 3D SEM, FEI Company, Hillsboro, OR, USA). The technical route diagram proposed for this paper is shown in Figure 1.

- In Tables 1 and 2, paragraphs 2 and 3 of Section 3.1, Sections 3.3, 3.4.2, 3.4.3, 4 and 5, and the legend of Figure 2, the word “building” has been changed to “construction”.
- In Section 2.2.2, the phrase “Geo-technical Test Specification (SL 237-020-1999)” has been changed to “highway geotechnical Test (JTG 3430-2020)”.
- In Section 3.1, the paragraph 1 is updated to following version:

It can be seen from Table 2 and Figures 2 and 3 that the strength growth rates in the table are all based on G8. The unconfined compressive strength of G8 solidified sludge without aggregate is 0.944 MPa. In general, the unconfined compressive strength of solidified sludge increases with the increase in particle size and content of construction debris, and the strength growth rate after adding construction debris is 8.50%~72.16%. The sample mixed with ordinary sand also has the same change trend. However, when the particle size is less than 0.25 mm, the unconfined compressive strength of solidified sludge is increased more by adding construction debris. Among them, the unconfined compressive strength of 1G8S5 is 1.024 MPa, while the unconfined compressive strength of sample 1G8S’5 is 0.861. The strength ratio of both is about 1.19. When the amount of construction debris is increased from 5% to 15%, the strength ratio is greater than 1 MPa. With the increase in aggregate size, the unconfined compressive strength of solidified sludge mixed with construction debris is about 90% of that of ordinary sand-solidified sludge. The results indicate that the porous surface structure of fine-sized construction aggregates adsorbs free water, thereby reducing the thickness of the water film surrounding sludge particles. Compared with ordinary sand of equivalent particle size, this aggregate type provides a more effective skeletal reinforcement due to its enhanced structural integrity. Furthermore, the incorporation of larger-sized construction aggregates substantially improves the unconfined compressive strength of the stabilized sludge. Collectively, these findings demonstrate that the use of construction aggregates as a skeletal matrix material is fundamentally viable.

- In Section 3.2, the phrase “wetting cycle” has been changed to “wetting cycling”.
- In Section 3.3, the sentence “It can be seen from the figure that the permeability coefficient of sample G8S10 is 2.00 × 10^−7^ cm/s, a permeability coefficient of 3.20 compared with sample G8 at 1 × 10^−8^ cm/s” has been changed to “As shown in the figure, the permeability coefficient of sample G8S10 is 2.00 × 10^−7^ cm/s, which is approximately 6.25 times higher than that of sample G8 (3.20 × 10^−8^ cm/s).”
- Figure 7 has been updated to following version:

**Figure 7:**
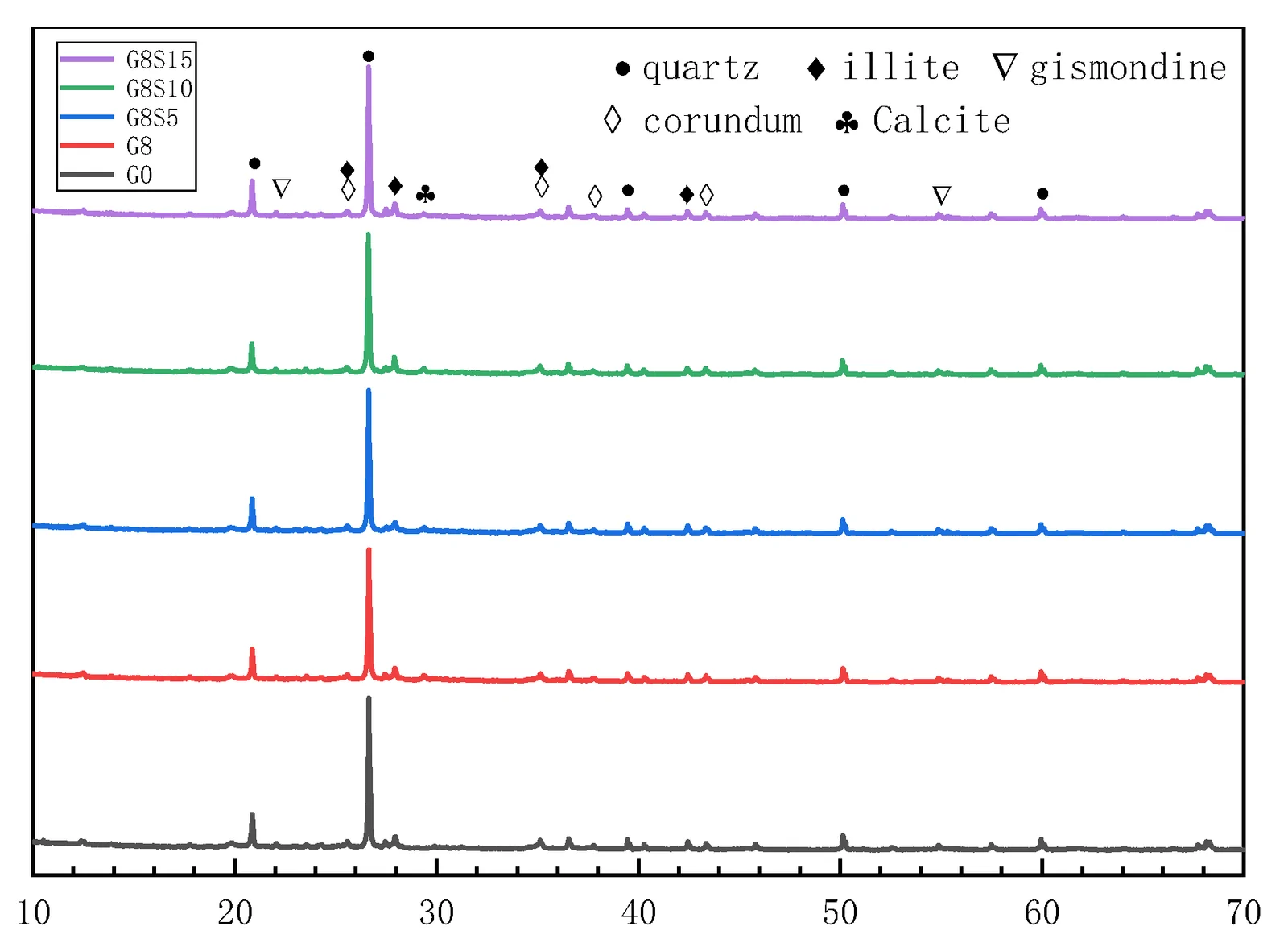
X-ray diffraction patterns of G0, G8, G8S5, G8S10 and G8S15.

- In Section 4, the sentence “where *i*, *j*, and *k* represent the numbers of sludge particles, cementitious aggregates, and construction debris particles within the unit cross-section, respectively.” is added after the equation “$\frac{\sum \sigma_{1i}+\sum \sigma_{2j}+\sum \sigma_{3k}}{i+j+k}$”.
- In Section 5, the sentence “X-ray diffraction pattern, mercury intrusion analysis and scanning electron microscope image analysis” has been changed to “XRD, MIP and SEM”; “89% to 119%” has been changed to “0.89 to 1.19”.
- The retracted reference [16] has been removed, and a new reference [22] has been added and cited in Section 2.1. With this correction, the order of some references has been adjusted accordingly.

16. Shanmugasundar, G.; Krishnan, G.S.; Babu, L.G.; Kumar, S.; Makos, M. Investigation of ferronickel slag powder for marine application by using MIP method. *Mater. Res. Express* **2022**, *9*, 055501. https://doi.org/10.1088/2053-1591/ac6abb.

22. *JTG 3430-2020*; Test Methods of Soils for Highway Engineering. China Communications Press: Beijing, China, 2020. (In Chinese)

The authors state that the scientific conclusions are unaffected. This correction was approved by the Academic Editor. The original publication has also been updated.

## References

[1] Shi Y., Weng H., Yu J., Gong Y. (2023). Study on Modification and Mechanism of Construction Waste to Solidified Silt. Materials.

